# Neuroprotective properties of *Linzia gerberiformis* aqueous leaves extract against aluminium chloride (AlCl_3_) -induced cognitive impairment: Involvement of cholinergic, GABAergic, and antioxidant mechanisms

**DOI:** 10.1016/j.ibneur.2026.02.007

**Published:** 2026-02-07

**Authors:** Jospin Chirac Noubouwo, Gwladys Temkou Ngoupaye, Steve Brunel Kenfack Ngoufack, Bibiane Tatiana Diebo Kom, Aurelien Fossueh Foutsop, Blesdel Maxwell Adassi, King-Ghislain Gnoupa, Francis Bray Yassi

**Affiliations:** a Department of Animal Biology, Animal Physiology and Phytopharmacology Research Unit, University of Dschang, Dschang, Cameroon; b Department of Biological Sciences, Faculty of Science, University of Maroua, Maroua, Cameroon; c Department of Biological Sciences, Faculty of Science, University of Ngaoundéré, Ngaoundéré, Cameroon

**Keywords:** *L. gerberiformis*, Aluminium chloride, Alzheimer's disease, Oxidative stress, Mice, Aqueous extract of leaves

## Abstract

Neurodegenerative diseases, such as Alzheimer's disease (AD), are characterized by oxidative imbalance, leading to memory deficits and cognitive impairments. Aluminium chloride (AlCl_3_), a neurotoxin found in certain foods and medications, disrupts neurotransmitter systems, thereby exacerbating cognitive decline. Current drug development strategies aim to counter these effects through cholinesterase inhibition, activation of GABAergic transmission, the use of antioxidants, and the promotion of neuroprotection. This work was conducted to assess the neuroprotective properties of the aqueous extract of *Linzia gerberiformis* (*L. gerberiformis*) leaves against AlCl_3_ induced cognitive impairment in mice. AlCl_3_ (70 mg/kg) was administered orally in mice to induced memory loss a core feature of Alzheimer Disease. Mice were pretreated with aqueous extract of *L. gerberiformis leaves* (75, 150 and 300 mg/kg) for six weeks, and memory integrity was assessed using the object location test (OLT) and the T-Maze test. One hour after completion of the T-Maze, the mice were sacrificed, the hippocampus and prefrontal cortex were then collected to assess the cholinergic (acetylcholinesterase (AChE) and acetylcholine (ACh)), GABAergic systems and oxidative stress (nitric oxide (NO), malondialdehyde (MDA) SOD, Catalase (CAT), reduced glutathione (GSH)). The aqueous extract of *L. gerberiformis* leaves demonstrated significant effects (P < 0.01 and P < 0.05) at doses of 75 and 150 mg/kg in improving short-term learning memory, as well as a significant enhancement (P < 0.05) of long-term spatial memory on day 3 at the dose of 75 mg/kg. AlCl_3_ (70 mg/kg) induced increase in AChE (P < 0.05; P < 0.01), NO (P < 0.05; P < 0.001), and MDA (P < 0.05; P < 0.01), while decreasing ACh (P < 0.01), GABA (P < 0.05; P < 0.01), SOD (P < 0.01; P < 0.001), CAT (P < 0.05), and GSH (P < 0.01) in the hippocampus and prefrontal cortex. Pretreatment with aqueous extract of *L. gerberiformis* leaves significantly restored these parameters (P < 0.01; P < 0.001) for Ach at the doses 75 and 150 mg/kg and GABA (P < 0.05) at all doses. Significant improvement was also observed for SOD (P < 0.05 and P < 0.01), GSH, and MDA (P < 0.05 and P < 0.01), as well as for NO (P < 0.05 and P < 0.01) in both the hippocampus and prefrontal cortex. The present study established that the aqueous extract of *L. gerberiformis* leaves ameliorated AlCl_3_-induced neurotoxicity by modulating the activation of the cholinergic, GABAergic and antioxidant pathways.

## Highlights

• AlCl_3_ induced cognitive impairment in mice.
• *L. gerberiformis* exhibits antioxidant activity *in vitro*.
• *L. gerberiformis* improves short-term and reference memory in behavioral tests.
• *L. gerberiformis* restores alterations in the cholinergic and GABAergic systems, as well as antioxidant markers in the hippocampus and prefrontal cortex.

## Introduction

Aging is characterized by a progressive decline in physiological capacities (López-Otín et al., 2013, Amarya et al., 2018), and its cerebral acceleration contributes to the onset of Alzheimer's disease (AD) (Liu, 2022). AD is a neurodegenerative condition that destroys GABAergic and cholinergic neurons, impairing synaptic information transmission (Patthy et al. 2021). This phenomenon manifests as a decline in cognitive functions, including attention, learning, and memory (Sikora et al., 2021, Fantuzzi, 2024). The impairment of these memory processes can be induced by various chemical substances, notably heavy metals such as lead, mercury, and aluminium chloride (AlCl_3_) (Brough and Jouhara, 2020, Dike et al., 2023). Chronic exposure to AlCl_3_, a potent biological inhibitor, is concerning due to its accumulation in tissues and often ineffective hepatic elimination (Nabipour et al. 2025). AlCl_3_ primarily enters the body through dust, food, and medications, accounting for 90 % of total exposure and causing cellular damage, particularly in the brain (Nabipour et al. 2025). Recent studies link this exposure to neurological symptoms and biochemical changes similar to AD in the cerebral cortex and hippocampus (Yisa et al. 2024). Exposure to AlCl_3_ results in its components binding to transferrin in the blood, allowing the complex to easily cross the blood-brain barrier (BBB) and accumulate primarily in the prefrontal cortex and parts of the hippocampus (Borai et al., 2017, Sedik et al., 2025). In these regions, AlCl_3_ induces oxidative stress by decreasing endogenous antioxidants in favor of oxidants, creating cytotoxicity at the root of AD (Dey and Singh, 2022). The compound reduces acetylcholine (Ach) synthesis and disrupts GABA function, leading to Aβ accumulation, Tau protein hyperphosphorylation, and glutamate excitotoxicity, followed by neuronal necrosis (Dey and Singh, 2022, Kazmi et al., 2024a). These effects, including increased acetylcholinesterase (AchE) activity and decreased cerebral GABA levels, cause memory loss similar to that observed in AD, making AlCl_3_ a primary neurotoxicity model in lab animals (Zhang et al. 2022). AD is a social and public health problem that deserves scientific attention, as it represents the sixth leading cause of death, with a prevalence of around 6.5 million suffering in the USA by 2022 and 50 million worldwide (Kumar et al., 2021, Rajan et al., 2021). The annual global cost of care is US$ 1000 billion distributed into to the high cost of available synthetic drugs such as acetylcholinesterase inhibitors (donepezil, galantamines, rivastigmine), N-methyl-D-Aspartate (NMDA) receptor antagonists like memantine (Breijyeh and Karaman, 2020), and intensive and costly care provided to poorly managed patients. Interestingly, the AD drugs available are unfortunately associated with side effects such as vomiting, diarrhea, nausea, insomnia, slowed heart rate and hepatotoxicity (Sharma, 2019, Chakraborty and Diwan, 2022). These side effects have lead to the medical needs to develop multi-target drugs from natural products with fewer undesirable side effects for local populations deemed with the fact that natural products such as plants have gained in popularity in recent years due to their accessibility and potency (Ayaz et al. 2024).

Plants such as *Vitis vinifera* (Borai et al. 2017), *Capparis sepiaria* (Yassi et al. 2023) and *Pluchea lanceolata* (Asteraceae) (Asirvatham et al. 2022) have been shown to have antiamnesic and neuroprotective properties through the modulation of antioxidants, regulation of cholinergic system and neuroinflammation. Despite the relevant results of these scientific studies, the incidence of AD continues to rise, and meaningful efforts are put in place to develop therapeutics strategies for neurodegeneration considering that the signalling pathways involved in AD’s physiopathology remain poorly understood.

*L. gerberiformis* (Oliv. &Hiern) H. Rob also known as *Vernonia glabra* (Steetz) Vatke or *Linzia glabra* is an herbaceous plant found in farmland in the Littoral and Western regions of Cameroon. Commonly known as ՙՙNdolet sucré՚՚, it is used in traditional medicine for the treatment of diabetes, wounds, gonorrhea, dysentery, coughs and gastrointestinal problems in Central and Southern Africa (Tsabang et al., 2016, Maroyi, 2021). Phytochemical compounds identified from this species include alkaloids, flavonoids, polyphenols, steroids and tannins (Kitonde et al., 2013, Maroyi, 2021, Wanna et al., 2023). Recent studies have reported its *L. gerberiformis* prevents cognitive deficits induced by the combined effects of ovariectomy and chronic unpredictable mild stress in rats (Noubouwo et al. 2025). We hypothesize that the aqueous extract of *L. gerberiformis* leaves exerts neuroprotective effects against AlCl_3_-induced memory impairment through antioxidant, cholinergic, and GABAergic mechanisms. This study sought to assess the preventive effect of the extract on memory deficits induced by AlCl₃ neurotoxicity. This includes investigating changes in acetylcholinesterase activity, acetylcholine and GABA levels in the hippocampus and prefrontal cortex, as well as evaluating the antioxidant properties of the extract and their contribution to neuroprotection.

## Materials and methods

### Drugs used

The following drugs were used in the study: aluminium chloride (Sigma-Aldrich, St. Louis, USA) and memantine (Sigma-Aldrich, St. Louis, USA).

### Plant material

*L. gerberiformis* leaves were collected in Dschang (Menoua Department, West Region) in the Likong-foréké locality in February 2022. One sample was authenticated at the Cameroon National Herbarium by comparison with sample N° 801859. The leaves were then washed (tap water), dried (away from the sun) and ground using a mill. Later, 300 g of the powder obtained was macerated in 3 l of distilled water for 24 h. To obtain the dry extract, the macerate was filtrated with whatman n°4 paper, and the filtrate was dried in oven at 40°C to have a 43.80 g dried extract (extraction yield was 14.6 %).

### Study of the in vitro antioxidant activity of the aqueous extract of *L. gerberiformis leaves*

#### DPPH method

The DPPH test methodology was developed by Mensor et al. (2001) to assess the antioxidant activity of the aqueous extract of *L. gerberiformis* leaves and vitamin C (L-ascorbic acid). Methanolic solutions of *L. gerberiformis* and vitamin C (2 mg/mL) were prepared in a 96-well plate, where 20 µL of methanol (98 %) were added to the wells in rows 2–8, and 20 µL of the *L. gerberiformis* solution were introduced into the first two rows of the first four columns, with successive dilutions to establish a concentration range from 2 mg/mL to 0.0156 mg/mL. Subsequently, 180 µL of DPPH methanolic solution (0.08 mg/mL) were added to each well in the first three columns, while 180 µL of methanol were added to the fourth column, bringing the total volume to 200 µL per well. The final concentrations of the extract ranged from 200 µg/mL to 1.56 µg/mL. The plates were incubated for 30 min at room temperature in the dark, after which optical densities were measured at 517 nm using a FLUOstar Omega microplate reader. Vitamin C served as a positive control, and DPPH alone was used as a control. The percentages of antioxidant activity for each sample were calculated using the following formula:

$$\mathrm{Antioxydant}\;\mathrm{activity}\;=\frac{\mathrm{DPPH}\;\mathrm{Absorbance}-\;\left(\mathrm{Essay}\;\mathrm{Absorbance}-\mathrm{Blank}\;\mathrm{Absorbance}\right)\;}{\mathrm{DPPH}\;\mathrm{Absorbance}}X100$$

Assay = sample + DPPH methanolic solution Blank = sample + methanol; DPPH = Control

The different percentages of antioxidant activity were used to determine the EC_50_ (the concentration of the sample that can scavenge 50 % of DPPH·) (Yassa et al., 2008).

### FRAP method (Ferric reducing antioxidant power)

The reducing power of the extract was determined according to the protocol described by Benzie and Strain (1996). The FRAP reagent was prepared by mixing a sodium acetate buffer solution (300 mM, pH 3.6), a solution of 2,4,6-tris(2-pyridyl)-1,3,5-s-triazine (TPTZ) (10 mM), and a solution of FeCl₃ in the proportions of 10:1:1. A volume of 5 µL of the sample (2 mg/mL) was mixed with 95 µL of the FRAP reagent. The mixture was incubated for 30 min at 37 °C in the dark. After incubation, the optical density was measured using a spectrophotometer (FLUOstar Omega microplate reader) at 593 nm. Vitamin C was used as a positive control. The reducing power of the sample was calculated from the calibration curve of the FeSO₄ solution, with concentrations ranging from 100 µmol/L, 50 µmol/L, 25 µmol/L, 12.5 µmol/L, 6.25 µmol/L, 3.125 µmol/L, 1.56 µmol/L, and 0.781 µmol/L, and was expressed as millimole equivalents of FeSO₄ per gram of sample.

### Animals

Mice with a body weight between 20 and 25 g and aged 8–10 weeks were reared to adulthood in the animal facility of the Animal Physiology and Phytopharmacology Research Unit, Department of Animal Biology, Faculty of Science, University of Dschang. These animals were raised at a constant temperature of 23 ± 1 °C in polyacrylic cages. Additionally, the subjects were exposed to a natural light-dark cycle of 12 h. In order to prevent mating, the subjects were separated into subgroups consisting of four males and three females. Furthermore, the mice were provided with unrestricted access to food and water while being maintained under standard laboratory conditions. This rearing process was conducted in accordance with NIH guidelines for the care and use of laboratory animals, as well as Directive 2010/63/EU on animal experiments. A series of measures were implemented with the objective of minimizing stress and pain in the animals.

### Experimental protocols

The administration of AlCl_3_ (70 mg/kg) provided an effective model for investigating the neurotoxicity and its impact on cognitive function, as described in (Ali et al. 2016). In this study, 42 adult mice were subsequently divided into six groups of seven mice each (four males and three females per group) (Yassi et al., 2023) and treated according to the protocol specified in (Abd El-Aziz et al., 2022). The test was carried out as a preventive treatment.

Group I: Vehicle, received distilled water (10 mL/kg/day) orally for 42 days;

Group II: Negative control, received distilled water (10 mL/kg/day) followed by AlCl_3_ (70 mg/kg/day) for 42 days;

Group III: positive control, received memantine (20 mg/kg/day) + AlCl_3_ (70 mg/kg/day) orally for 42 days;

Groups IV, V and VI: received *L. gerberiformis* aqueous leaf extract at doses of 75, 150 and 300 mg/kg/day respectively + AlCl_3_ (70 mg/kg/day) orally for 42 days.

**Fig. 1:**
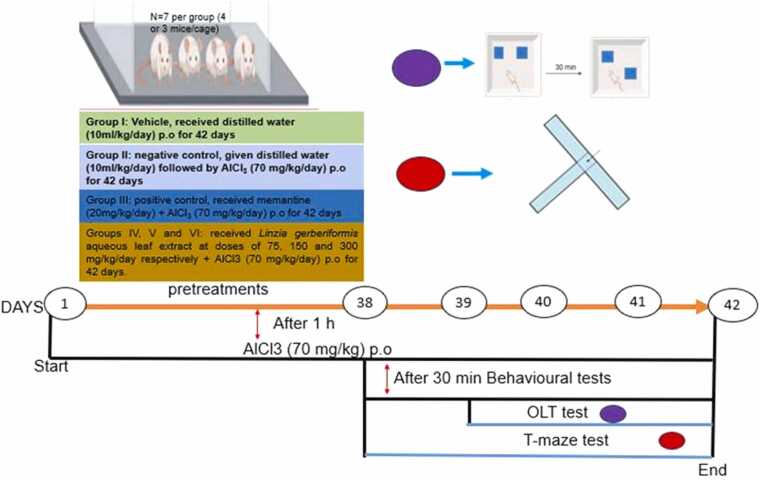
Timeline of drug administration and behavioral assessments in mice.

Mice were pretreated with distilled water (vehicle), memantine (positive control) and the aqueous extract of *L. gerberiformis* leaves at different doses (75, 150, 300 mg/kg) one hour prior to the administration of AlCl_3_ (70 mg/kg/day) orally from day 1–42. After the administration of AlCl_3_ (70 mg/kg/day), animals waited 30 min, then the OLT familiarization phase was carried out from day 39–41 followed by the actual test on day 42. Similarly, the T-Maze test began on day 38 and end on day 42 (Fig. 1). The Object Location Test (OLT) and T-Maze were performed with a one-hour interval between tests. The two behavioral tests were performed according to the following protocols. The behavioral tests were performed between 9 am to 5 pm.

### Behavioral test

#### Object location test (OLT)

The object location task assesses hippocampal spatial memory and measures the animal's ability to discriminate the change in position of an object (Ennaceur et al. 1997). Two wooden cubes were used as objects and placed in a square wooden box (40 × 40 ×45 cm). The mice were first familiarized by introducing them individually into the wooden box without any objects and letting them explore for 15 min, over three successive days. During the actual test phase on day 42, the rats were placed individually in the wooden box containing two identical wooden cubes for 5 min. After a latent period of 30 min, the test phase began, which involved shifting the position of an object to a new location. The period spent exploring objects in familiar and new location lasted 5 min. Intact memory was supported by the fact that the animals took longer to explore the object in the new location (Antunes and Biala, 2012).

### T-maze neurobehavioral test

The T-maze is a T-shaped device generally used in behavioral neuroscience to assess spatial working memory in rodents (Deacon and Rawlins, 2006). The T-shaped device comprises two opposing arms of identical size (50 cm long, 10 cm wide and 25 cm high walls) and a starting arm (70 cm long, 10 cm wide and 25 cm high walls) separated from the maze by a door. At the end of the two opposite arms was placed a small cup containing 45 mg of food. The test was carried out in two phases, the first of which was the habituation (familiarization with reward), lasting 15 min over the first three days. The second phase, training, consists of ten trials, with an interval of 5 min between trials for the last three days. The first trial, called the forced trial, consists in randomly choosing an arm, then blocking it with a sliding door and placing the reward in the opposite arm, leaving the animal no choice but to choose the arm with the reward. In the last 9 trials, both arms will be opened, but the reward will be given in the arm that did not receive the reward in the previous trial. When two trials follow one another, on the second trial the rodent tends to choose the arm not previously visited, reflecting the memory of the first choice. This is known as "rewarded spontaneous alternation". An entry was recorded when the animal is completely in the arm, including the tip of its tail. If the animal selects the arm not containing the reward on the second trial, the experimenter gives it a chance to change its choice by keeping the arm containing the reward. Entry into the arm visited on the previous trial will be recorded as a working memory error. The percentage of correct choices will be calculated according to the formula opposite % correct choice = (correct choices/Total trials) x 100 (Bezu et al. 2017). The AlCl_3_ (70 mg/kg/day) was administered 30 min before training, and the different pretreatment administered one hour prior to AlCl_3_ administration.

### Animal sacrifice, sample collection

At the end of the behavioral assessments on day 42, the animals were sacrificed by decapitation, and the skull was opened to remove the prefrontal cortex and hippocampus. These tissues were placed into Eppendorf tubes, weighed, vacuum-sealed, and stored at −20°C for subsequent processing. The tissues were homogenized using 0.1 M phosphate buffer (pH 7.4) (Cat. No. P4417, Sigma-Aldrich, USA) containing 1 % (w/v) Triton X-100 (Cat. No. T8787, Sigma-Aldrich, USA). The resulting homogenate was then centrifuged at 3000 rpm for 15 min. Finally, the supernatant was collected for subsequent biochemical assays (Ngoupaye et al. 2018).

### Biochemical analysis

#### Determination of acetylcholinesterase (AChE) level in the prefrontal cortex and hippocampus

AChE activity was measured according to Ellman et al. (1961). Briefly, 1000 µL of phosphate buffer (Cat. No P4417, Sigma-Aldrich, USA), 100 µL of 5,5-dithiobis-2-nitrobenzoic acid (DTNB) (Cat. No D8130, Sigma-Aldrich, USA), 25 µL of acetylthiocholine iodide (Cat. No. 01480, Sigma-Aldrich, USA), and 10 µL of homogenate were mixed. Absorbance readings were taken at 412 nm at one, three, and five minutes using a BIORAD spectrophotometer (SmartSpec 3000, Cat. No. 170–2501, USA). In the blank tube, the homogenate was replaced with the grinding solution, and 1000 µL of phosphate buffer, 100 µL 5,5-dithiobis-2-nitrobenzoic acid (DTNB), and 25 µL of acetylthiocholine iodide were added. Enzyme activity was expressed as nmol/minutes/mg protein; with protein levels determined using a commercially available standard kit (Dutch Diagnostics).

### Determination of acetylcholine (ACh) level in the Prefrontal cortex and hippocampus

For the evaluation of tissue acetylcholine (ACh) levels, samples (100 μL) and a standard acetylcholine chloride solution (100 μL) (Cat. No. A6625, Sigma-Aldrich, USA) were mixed with 200 μL of alkaline hydroxylaminutese (Cat. No. H438227, Sigma-Aldrich, USA). The mixture was stirred and allowed to stand for two minutes. After this period, 100 μL of one-third diluted HCl and 100 μL of FeCl3 (Cat. No. F157740, Sigma-Aldrich, USA) were added, and the absorbance was immediately measured at 540 nm using a Bio-Rad spectrophotometer (SmartSpec 3000, Cat. No. 170–2501, USA). Results were reported in M/mL. In the blank tube, the homogenate was replaced with the grinding solution, and the reagents were added in the following order: one-third diluted HCl, alkaline hydroxylamine, and FeCl_3_ (Hestrin, 1949).

### Determination of Gamma-aminubutyric acid (GABA)

The measurement of gamma-aminobutyric acid (GABA) in the hippocampus and prefrontal cortex of mice was performed using a colorimetric technique as described by Moto et al. (2018). In an alkaline medium, GABA reacted with ninhydrin in the presence of glutamate to produce a detectable coloration at 451 nm, proportional to its concentration. Reagents were added in the following order: ninhydrin (Cat. No. NX0403–1, Sigma-Aldrich, USA), 10 % TCA (Cat. No. T6399, Sigma-Aldrich, USA), GABA standard solution (Cat. No. A2129, Sigma-Aldrich, USA), glutamate (Cat. No. PHR2634, Sigma-Aldrich, USA), and the sample to be analyzed. After incubation at 60°C for 30 min, followed by incubation at 25°C with copper tartrate (Cat. No. T514071, Sigma-Aldrich, USA), the absorbance of the supernatant was measured. A blank, which contained the same reagents as the samples except that the homogenate was replaced with the grinding solution, was used for calibration. Absorbance was measured using a BIORAD spectrophotometer (SmartSpec 3000, Cat. No. 170–2501, USA). This method enabled precise quantification of GABA in brain tissue.

### In vivo antioxidant activity of *L. gerberiformis* aqueous leaf extract

#### Assay of superoxide dismutase

This assay evaluates the ability of superoxide dismutase (SOD) to inhibit the auto-oxidation of adrenaline to adenochrome in a basic environment. A carbonate buffer at pH 10.2 (880 µL) and homogenate (70 µL) were combined in a tube. Subsequently, 100 µL of 0.3 mM adrenaline was added, and the timer was initiated. Absorbance was measured using a BIORAD spectrophotometer (SmartSpec 3000, Cat. No. 170–2501, USA) at 60, 120, and 180 s, compared to the blank (which received the same solutions as the samples, except that the homogenate was replaced with the grinding solution). SOD activity was reported in units per mg of protein (Dimo et al. 2006).

#### Assessment of catalase activity

Catalase catalyzes the decomposition of hydrogen peroxide, leading to the formation of an unstable blue-green precipitate of perchloric acid in the presence of potassium dichromate. In test tubes, 50 µL of homogenate was sequentially added to 750 µL of phosphate buffer (0.1 M, pH 7.5). The stopwatch was started immediately after adding 200 µL of hydrogen peroxide (50 mM) (Cat. No. H1009, Sigma-Aldrich, USA). After one minute, the reaction was stopped by adding 1000 µL of a 5 % potassium dichromate and acetic acid solution. For the blank tube, 50 µL of the grinding solution and 750 µL of phosphate buffer (0.1 M, pH 7.5) were used. All tubes were then incubated in boiling water for 10 min. After cooling, the optical density was measured at 570 nm using a BIORAD spectrophotometer (SmartSpec 3000, Cat. No. 170–2501, USA) against the blank (which contained the same solutions as the samples, except the homogenate was replaced with the grinding solution) (Sinha, 1972). The remaining amount of hydrogen peroxide after the addition of perchloric acid was determined using a calibration curve.

### Measurement of reduced glutathione levels

Tissue levels of reduced glutathione (GSH) were determined using the method described by Ellman (1959). Briefly, 100 µL of homogenate was added to tubes containing 1.5 mL of Ellman’s reagent (Cat. No. D8130, Sigma-Aldrich, USA) and 100 µL of phosphate buffer solution. The mixture was incubated at room temperature for one hour. Absorbance was measured at 412 nm using a spectrophotometer (SmartSpec 3000, Cat. No. 170–2501, USA) against a blank, which contained the same reagents as the samples except that the homogenate was replaced with the grinding solution. Tissue GSH content was expressed in µmol/mL/mg of protein. Optical density (OD) readings at 412 nm were used to calculate the concentration of reduced glutathione (GSH).

### Determination of malondialdehyde levels

Malondialdehyde (MDA), the end product of lipid peroxidation, was quantified in tissue samples using spectrophotometer according to the method described by Hritcu et al. (2011). Briefly, 200 µL of homogenate was added to tubes containing 1 mL of 50 % trichloroacetic acid diluted in 0.1 M HCl and 1 mL of 26 mM thiobarbituric acid. The tubes were then tightly sealed and incubated at 90 °C for 30 min. After cooling, the mixture was centrifuged at 3000 rpm for 10 min to separate the organic layer. Absorbance was immediately measured at 532 nm using a spectrophotometer (SmartSpec 3000, Cat. No. 170–2501, USA) against the blank. MDA levels were expressed as nmol per mg of tissue.

### Determination of nitric oxide levels

In the spectrophotometer cuvette, 200 µL of tissue homogenate and 200 µL of a 1 % sulfanilamide solution (prepared in 5 % phosphoric acid; Cat. No. C994Z77, Thomas Scientific) were added. After mixing, the sample was protected from light for 5 min. Subsequently, 200 µL of 1 % naphthylethylenediaminutese (Cat. No. C994Z45, Thomas Scientific), prepared in a Tris-hydroxymethylaminomethane buffer, was added to the reaction mixture. The sample was kept in the dark for an additional 5 min. Optical densities (OD) were measured within 30 min following incubation at a wavelength of 546 nm. The concentration of NO was determined using the calibration curve equation, established from various concentrations of NaNO₂ (Zhang et al., 2017).

### Statistical analysis

The data were analyzed using GraphPad Prism version 8.4.2 statistical software and expressed as mean ± SEM (Standard Error of the Mean). The Normal distribution of data was assessed using the Shapiro-Wilk normality test. When the data showed normal distribution, parametric tests were used One-way ANOVA followed by the Newman-Keuls multiple comparisons post-test was used to analyze discrimination index data (OLT), T-maze learning, and biochemical assay results. Two-way ANOVA followed by Bonferroni post hoc testing was employed to analyze OLT exploration time data. All possible comparisons between study groups were taken into account. Differences were considered statistically significant at p < 0.05.

## Results

### *In vitro* antioxidant activity of *L. gerberiformis* aqueous leaf extract

Table 1 shows the EC_50_ values for the aqueous extract of *L. gerberiformis* and vitamin C for DPPH radical scavenging. The EC_50_ value for vit C was 2.29 ± 0.33 μg/mL, whereas the EC₅₀ value for the aqueous extract of *L. gerberiformis* leaves was 195.88 ± 0.45 μg/mL. However, the aqueous extract of *L. gerberiformis* leaves exhibited ferric reducing activity with a value of 89.76 ± 0.33 mmol FeSO₄/g, compared to vitamin C's value of 173.85 ± 0.43 mmol FeSO₄/g.

**Table 1:** DPPH and FRAP antioxidant test.

**Samples**	**DPPH test**	**FRAP test**
	**EC**_**50**_**(µg/mL)**	**(mmol FeSO**_**4**_**/g)**
***L. gerberiformis***	195.88 ± 0.45	89.76 ± 0.33
**Vit C**	2.29 ± 0.33	173.85 ± 0.43

**Legend:** DDPH = 2,2-Diphenyl-2-picrylhydrazyl; FRAP= Ferric reducing antioxidant power

### Effect of *L. gerberiformis* aqueous extract treatment on behavioral tests in mice exposed to AlCl_3_ (70 mg/kg)

#### *L. gerberiformis* improve*s* memory in the object location test (OLT)

**Fig. 2:**
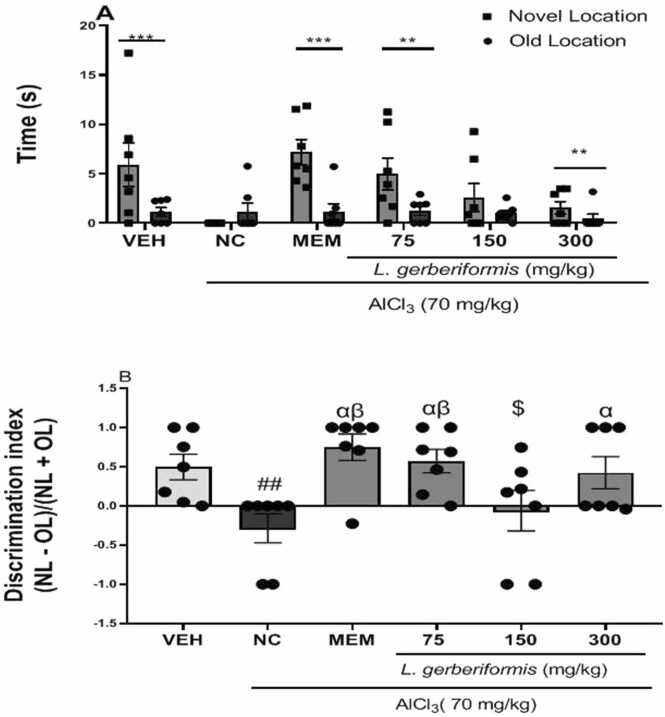
Effect of the aqueous extract of *L. gerberiformis* leaves on the object location test. A: Exploration duration between the locations; B: The discrimination index. Each bar represents the mean ± SEM (n = 7). ^**^P < 0.01; ^***^P < 0.001: significant difference from Old location in each group; ^α^P < 0.05, ^β^P < 0.01: significant difference from negative control; ##P < 0.01, significant difference compared to vehicle. $P < 0.05, significant difference compared to MEM. VEH: vehicle; NC: negative control. MEM: Memantine (20 mg/kg). The data were analyzed using a one-way ANOVA followed by a Newman-Keuls post hoc test (p < 0.05). The two bars shown in the figure at doses of 75 mg/kg and 150 mg/kg represent a comparison of the exploration time of the novel object relative to the familiar object.

Fig. 2 illustrates the duration of mice exploring objects. Fig. 2a shows the effect of different treatments on spatial location memory. A significant treatment effect was observed in the exploration time of the novel location compared to the old location among the groups [F(5, 72) = 3.746, P = 0.0045]. Animals treated with AlCl_3_ (70 mg/kg) spent more time exploring the old location than the novel location, although this difference was not significant. In contrast, pretreatment with *L. gerberiformis significantly* reversed the time spent exploring the novel location compared to the old location at doses of 75 mg/kg [F(1, 12) = 14.72, P = 0.0024] and 300 mg/kg [F(1, 12) = 6.016, P = 0.0304].

Fig. 2b shows a significant treatment effect on the discrimination index [F(5, 36) = 4.388, P = 0.0032]. Treatment with AlCl_3_ and distilled water led to a significant decrease in the discrimination index in the negative control group compared to the vehicle group [F(2, 18) = 6.465, P = 0.0077]. In contrast, memantine [F(2, 18) = 11.74, P = 0.0005], as well as *L. gerberiformis* at doses of 75 mg/kg [F(2, 18) = 9.467, P = 0.0029] and 300 mg/kg [F(2, 18) = 4.276, P = 0.0302], significantly reversed this effect. Compared to memantine, only the 150 mg/kg dose resulted in a significant decrease [F (2, 18) = 4.067, P = 0.0349] in the discrimination index (Fig. 2a).

### The aqueous extract of *L. gerberiformis* improves short-term spatial learning and reference memory in mice exposed to AlCl_3_ in the *T-maze*

**Fig. 3:**
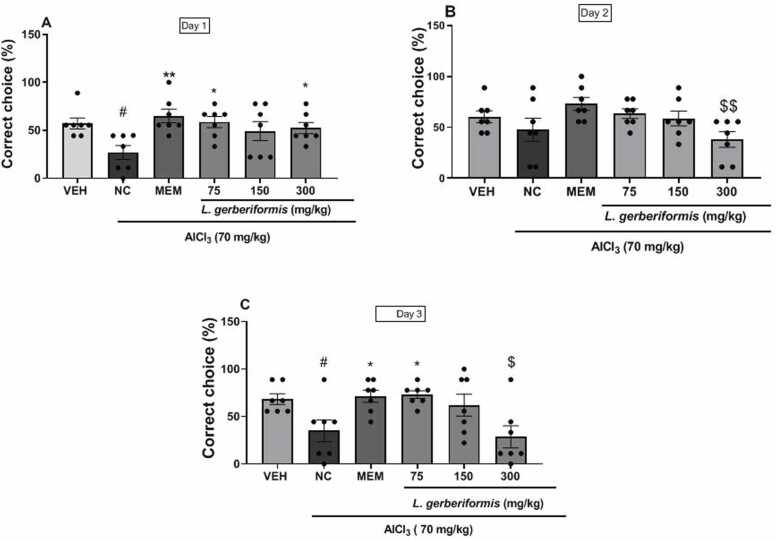
Effect of *L. gerberiformis* aqueous extract on spatial and reference learning memory in *T-Maze.* A: Represents Day 1; B: Represents Day 2; C: Represents Day 3. Each bar represents the mean ± SEM (n = 7). *P < 0.05; **P < 0.01: significant difference compared to negative control. #P < 0.05, significant difference compared to vehicle. $P < 0.05; $$P < 0.01: significant difference compared to MEM. VEH: vehicle; NC: negative control. MEM: Memantine (20 mg/kg). The data were analyzed using a one-way ANOVA followed by a Newman-Keuls post hoc test (p < 0.05).

The effect of the different treatments on the percentage of correct choices is illustrated in Fig. 3. A significant variation in the number of correct choices was observed on day 1 [F (4, 30) = 3.898, P = 0.0115], day 2 [F (4, 30) = 3.110, P = 0.0297], and day 3 [F (4, 30) = 4.748, P = 0.0044] in all animal groups. A significant decrease in the percentage of correct choices was observed on days 1 and 3 for the negative control group compared to the vehicle group ([F (2, 18) = 3.759, P = 0.0432] and [F (2, 18) = 6.637, P = 0.0069], respectively). In contrast, animals that received a pretreatment of aqueous extract of *L. gerberiformis* leaves at a dose of 75 mg/kg or 300 mg/kg exhibited a substantial enhancement in the percentage of correct choices on Day 1 ([(F (2, 18) = 7.266, P = 0.0049] and [F (2, 18) = 5.376, P = 0.0148]), respectively, as compared to the negative control group (Fig. 3a). On the third day of the experiment, the aqueous extract of *L. gerberiformis* leaves demonstrated a significant increase in the percentage of correct choices at a dose of 75 mg/kg. This increase was statistically significant compared to the negative control group (F (2, 18) = 5.174, P = 0.0168) (Fig. 3c). Memantine (20 mg/kg) demonstrated a substantial increase in the percentage of correct choices on day 1 [F (2, 18) = 9.385, P = 0.0016] and day 3 [F (2, 18) = 4.384, P = 0.0281] compared to the negative control. However, a non-significant decrease in the percentage of correct choices was also observed on day 2 in animals in the negative control group compared to the vehicle (Fig. 3b). The treatments resulted in a non-significant increase in this percentage. On days 2 and 3, the 300 mg/kg dose showed a significant decrease ([F (2, 18) = 7.806, P = 0.0036]; [F (2, 18) = 5.943, P = 0.0104]) in the percentage of correct choices compared to memantine.

### The aqueous extract of *L. gerberiformis* leaves reduces acetylcholinesterase (AChE) activity in the hippocampus and prefrontal cortex

**Fig. 4:**
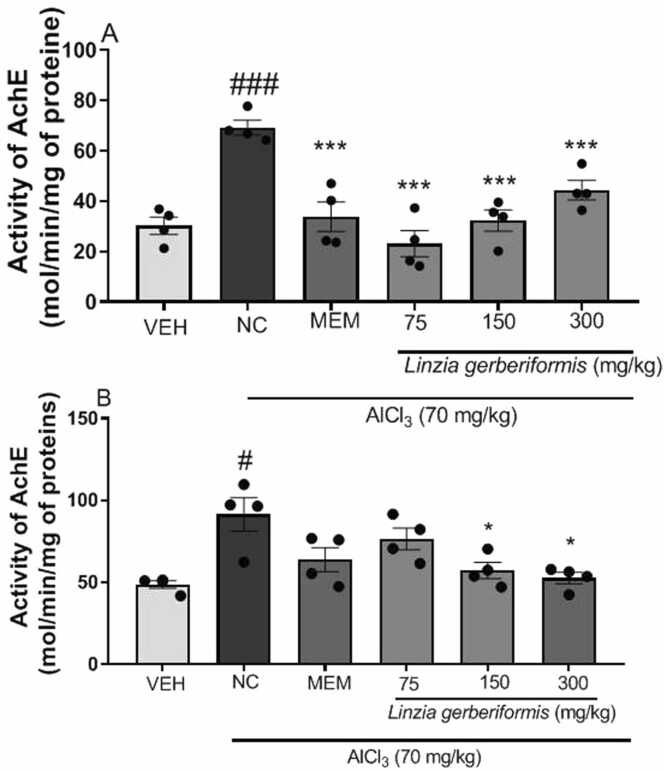
Effect of *L. gerberiformis* aqueous extract on acetylcholinesterase activity. A: Hippocampus; B: Prefrontal cortex. Each bar represents the mean ± SEM (n = 4). *P < 0.05, ***P˂0.001: significant difference from negative control. #p < 0.05; ###P < 0.001: significant difference compared to vehicle. VEH: vehicle; NC: negative control. MEM: Memantine (20 mg/kg). The data were analyzed using a one-way ANOVA followed by a Newman-Keuls post hoc test (p < 0.05). The data were analyzed using a one-way ANOVA followed by a Newman-Keuls post hoc test (p < 0.05).

Fig. 4 illustrates the effect of *L. gerberiformis* on acetylcholinesterase (AChE) activity in the hippocampus and prefrontal cortex.

Animals in the negative control group exhibited a significant increase in hippocampal AChE activity compared to the vehicle group [F (2, 9) = 51.83, P < 0.0001]. *L. gerberiformis* exhibited a decline in AChE levels at all administered doses, 75 mg/kg ([F (2, 9) = 47.50, P < 0.0001], 150 mg/kg [F (2, 9) = 38.85, P < 0.0001], and 300 mg/kg [F (2, 9) = 19.28, P = 0.0006]). Memantine also decreased AChE activity [F (2, 9) = 24.37, P = 0.0002] (Fig. 4a).

As illustrated in Fig. 4b, the negative control group exhibited an increase in AChE activity in the prefrontal cortex compared to the vehicle group [F (2, 9) = 8.604, P = 0.0082]. Pretreatment with the aqueous extract of *L. gerberiformis* leaves led to a decrease in AChE levels at doses of 150 mg/kg [F (2, 9) = 5.092, P = 0.0332] and 300 mg/kg [F (2, 9) = 6.885, P = 0.0153]. Memantine demonstrated a non-significant tendency to reduce AChE activity. At all doses, the aqueous extract of *L. gerberiformis* leaves showed a nonsignificant decrease in AChE compared with memantine (20 mg/kg) in the hippocampus and prefrontal cortex, respectively: [F(3,12) = 3.218, P = 0.0614] and [F(3,12) = 3.228, P = 0.0610].

### The aqueous extract of *L. gerberiformis* leaves increases acetylcholine (Ach) in the hippocampus and prefrontal cortex of mice exposed to AlCl_3_

**Fig. 5:**
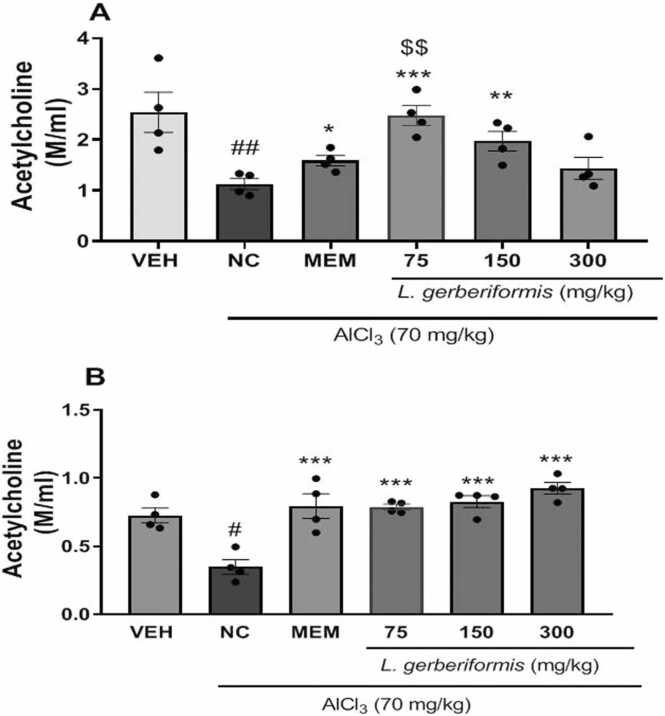
Effect of *L. gerberiformis* aqueous extract on acetylcholine concentration A: Hippocampus; B: Prefrontal cortex. Each bar represents the mean ± ESM (n = 4). *P < 0.05, **P < 0.01, ***P˂0.001: significant difference from negative control. #P < 0.05, ##P < 0.01: significant difference compared to vehicle. $$P < 0.01: significant difference compared to MEM. VEH: vehicle; NC: negative control. MEM: Memantine (20 mg/kg). The data were analyzed using a one-way ANOVA followed by a Newman-Keuls post hoc test (p < 0.05).

Fig. 5 shows the ACh levels in the hippocampus and prefrontal cortex of mice exposed to AlCl_3_.

Fig. 5a shows that daily exposure to AlCl_3_ in mice treated with the aqueous extract of *L. gerberiformis* leaves for six weeks resulted in a significant decrease in the ACh level in the hippocampus [F (2, 9) = 11.09, P = 0.0037] compared to the vehicle group. Pretreatment with the aqueous extract of *L. gerberiformis* leaves at the doses of 75 mg/kg [F(2, 9) = 28.73, P = 0.0001] and 150 mg/kg [F(2, 9) = 11.62, P = 0.0032] significantly increased ACh levels. Memantine significantly increased the ACh level [F (2, 9) = 6.122, P = 0.0210] compared to the negative control group.

The negative control group showed a significant decrease in the ACh level in the prefrontal cortex [F (2, 9) = 16.14, P = 0.0011] compared to the vehicle group. Pretreatment with the aqueous extract of *L. gerberiformis* leaves restored ACh levels at all doses (75 mg/kg [F(2, 9) = 30.95, P < 0.0001], 150 mg/kg [F(2, 9) = 29.64, P = 0.0001], and 300 mg/kg [F(2, 9) = 43.29, P < 0.0001]), compared to animals in the negative control group. Memantine significantly increased the ACh level [F(2, 9) = 14.40, P = 0.0016] compared to the negative control group. Compared with memantine, a dose of 75 mg/kg significantly increased acetylcholine concentration in the hippocampus only [F(2,9)= 8.924, P = 0.0073] (Fig. 5b).

### The aqueous extract of *L. gerberiformis* leaves increases GABA levels in the hippocampus and prefrontal cortex of mice exposed to AlCl_3_

**Fig. 6:**
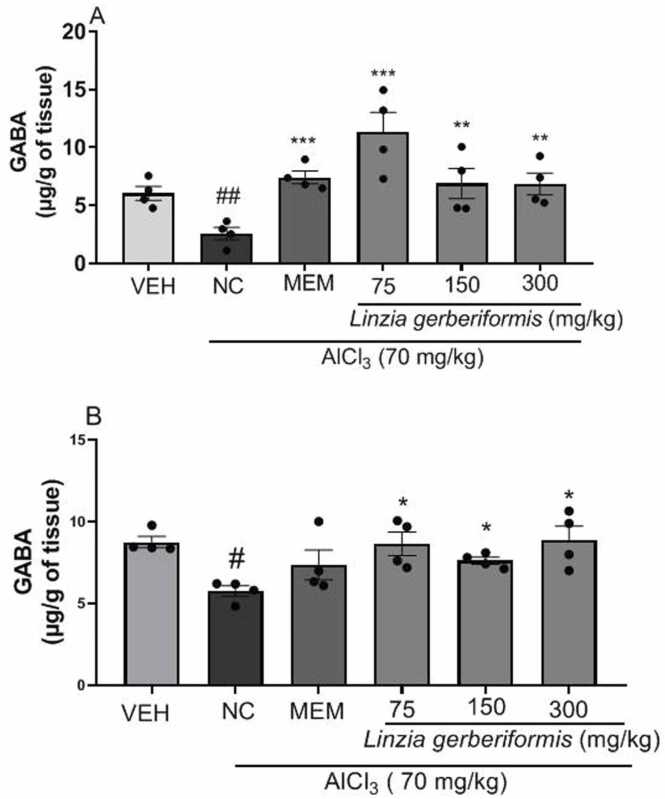
Effect of *L. gerberiformis* aqueous extract on GABA levels in hippocampus and prefrontal cortex. A: Hippocampus; B: Prefrontal cortex. Each bar represents the mean ± SEM (n = 4). *P < 0.05, ***P < 0.001: significant difference from negative control #p < 0.05, ##p < 0.01: significant difference from vehicle. VEH: vehicle; NC: negative control MEM: Memantine. The data were analyzed using a one-way ANOVA followed by a Newman-Keuls post hoc test (p < 0.05).

Fig. 6 shows the GABA levels in the hippocampus and prefrontal cortex of mice exposed to AlCl_3_.

Fig. 6a show that daily exposure to AlCl_3_ in mice treated with the aqueous extract of *L. gerberiformis* leaves for 42 days resulted in a significant decrease in the GABA level in the hippocampus [F (2, 9) = 42,08, P < 0.0001] compared with the vehicle group. Pretreatment with the aqueous extract of *L. gerberiformis* leaves at the doses of 75 mg/kg [F (2, 9) = 13,98, P = 0.0017], 150 mg/kg [F (2, 9) = 4264, P = 0.0498], and 300 mg/kg [F (2, 9) = 8.375, P = 0.0088] significantly increased GABA levels compared to the negative control group. Memantine significantly increased the GABA level in the hippocampus [F (2, 9) = 15.45, P = 0.0012] compared to the negative control group.

The negative control group showed a significant decrease in the GABA level in the prefrontal cortex [F (2, 9) = 13.36, P = 0.0020] compared to the vehicle group. Pretreatment with the aqueous extract of *L. gerberiformis* leaves restored GABA levels at all doses (75 mg/kg [F (2, 9) = 17.70, P = 0.0008], 150 mg/kg [F (2, 9) = 42.73, P < 0.0001], and 300 mg/kg [F (2, 9) = 8.898, P = 0.0074]) compared to animals in the negative control group.

### Effect of the aqueous extract of *L. gerberiformis* leaves on endogenous antioxidants (SOD, catalase and GSH) in the hippocampus and prefrontal cortex of mice

**Fig. 7:**
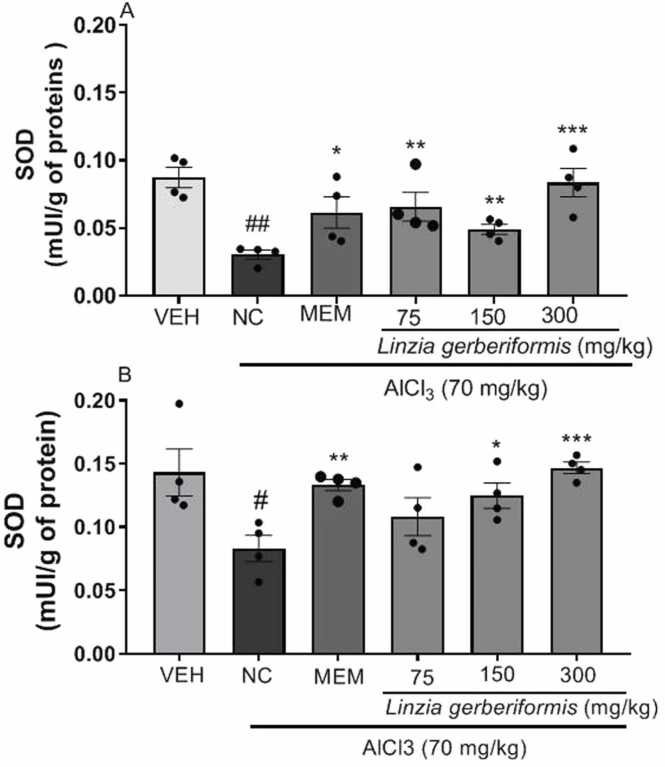
Effect of the aqueous extract of *L. gerberiformis* on the levels of SOD in hippocampus and prefrontal cortex. A: Hippocampus; B: Prefrontal cortex. Each bar represents the mean ±SEM (n = 4). *P < 0.05, **P < 0.01, ***P˂0.001 compared to NC. #p < 0.05, ##p < 0.01 compared to VEH. MEM = Memantine (20 mg/kg). VEH: Vehicle; NC: negative control. The data were analyzed using a one-way ANOVA followed by a Newman-Keuls post hoc test (p < 0.05).

Fig. 7 illustrates the SOD levels in the hippocampus and prefrontal cortex of mice after AlCl_3_ exposure.

The histograms in Fig. 7a show that daily AlCl_3_ administration to mice treated with *L. gerberiformis* leaf aqueous extract for 42 days significantly decreased SOD levels in the hippocampus [F(2, 9) = 35.62, P < 0.0001] compared to the vehicle group. Conversely, mice pretreated with the aqueous extract of *L. gerberiformis* leaves at 75 mg/kg [F(2, 9) = 21.89, P = 0.0003], 150 mg/kg [F(2, 9) = 8.304, P = 0.0090], and 300 mg/kg [F(2, 9) = 12.79, P = 0.0023] experienced a significant increase in hippocampal SOD levels compared to the negative control group. Memantine also significantly increased SOD levels in the hippocampus [F(2, 9) = 6.170, P = 0.0205] relative to the negative control group.

Compared to the vehicle group, the negative control group exhibited decreased SOD levels in the prefrontal cortex [F(2, 9) = 7.309, P = 0.0130]. The pretreatment with the aqueous extract of *L. gerberiformis* leaves increased SOD levels at 150 mg/kg [F(2, 9) = 5.505, P = 0.0274] and 300 mg/kg [F(2, 9) = 17.16, P = 0.0009] compared to the negative control group. Memantine (20 mg/kg) increased SOD levels in the prefrontal cortex [F(2, 9) = 10.67, P = 0.0042] compared to the negative control group (Fig. 7b).

**Fig. 8:**
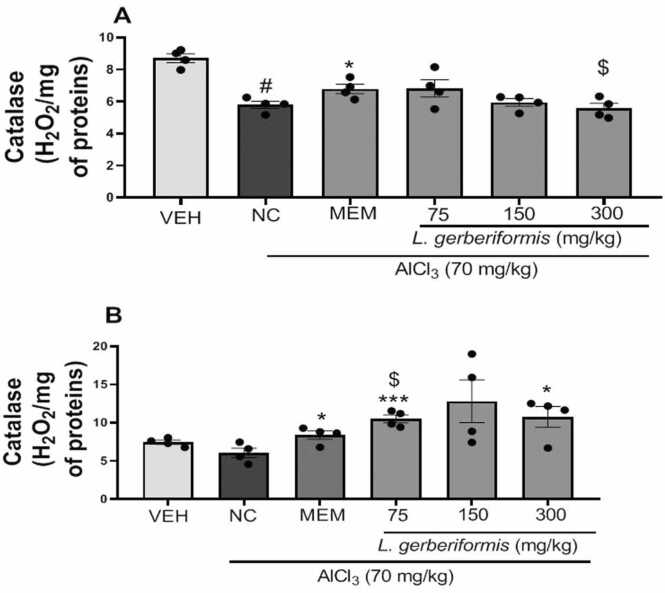
Effect of the aqueous extract of *L. gerberiformis* on the catalase activity in hippocampus and prefrontal cortex. A: Hippocampus; B: Prefrontal cortex. Each bar represents the mean ± SEM (n = 4). *P < 0.05, **P < 0.01, ***P˂0.compared to NC. ##p < 0.01, ###p < 0.001 compared to VEH. $p < 0.05 compared to MEM. MEM = Memantine (20 mg/kg). VEH: Vehicle; NC: negative control. The data were analyzed using a one-way ANOVA followed by a Newman-Keuls post hoc test (p < 0.05).

Fig. 8 depicted variations in catalase activity in the hippocampus and prefrontal cortex. The negative control group exhibited a significant decrease in catalase activity in the hippocampus [F (2, 9) = 48.29, P < 0,0001] compared to the vehicle group. Pretreatment with the aqueous extract of *L. gerberiformis* leaves at the doses (75 mg/kg [F (2, 9) = 1.690, P = 0.2382], 150 mg/kg [F (2, 9) = 0.4785, P = 0.1514] resulted in a non-significant increase in catalase activity in the hippocampus compared to the negative control group. Memantine significantly increased catalase activity [F (2, 9) = 5.389, P = 0.0289] compared to the negative control group (Fig. 8a).

Fig. 8b shows that daily exposure to AlCl_3_ for six weeks significantly decreased catalase activity [F (2, 9) = 3.567, P = 0,0723] in the prefrontal cortex compared to the vehicle group. Pretreatment with the aqueous extract of *L. gerberiformis* leaves resulted in a significant increase in catalase activity at doses of 75 mg/kg [F (2, 9) = 21.41, P = 0.0004] and 300 mg/kg [F (2, 9) = 5.363, P = 0.0293] compared to the negative control group. Memantine significantly increased catalase activity [F (2, 9) = 5.484, P = 0.0277] compared to the negative control group. Aqueous extract at a dose of 300 mg/kg produced a significant decrease in CAT activity in the hippocampus compared with memantine [F(2,9) = 5.267, p = 0.0306]. In the prefrontal cortex, the 75 mg/kg dose showed a significant increase in CAT activity compared to the group that received it [F (2, 9) = 5.366, P = 0.0292].

**Fig. 9:**
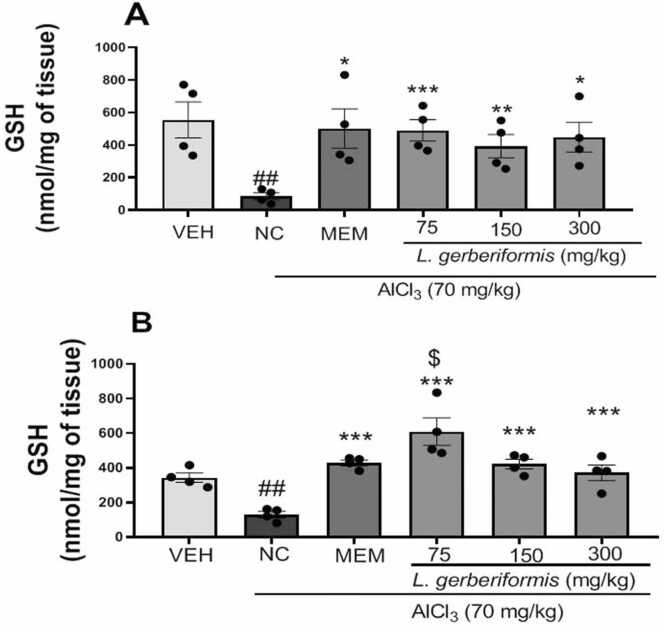
Effect of the aqueous extract of *L. gerberiformis* on the GSH in hippocampus and prefrontal cortex. A: Hippocampus; B: Prefrontal cortex. Each bar represents the mean ± SEM (n = 4). *P < 0.05, **P < 0.01, ***P˂0.001 compared to NC. ##P < 0.01, ###p < 0.001 compared to VEH. $P < 0.05 compared to MEM. MEM = Memantine (20 mg/kg). VEH: Vehicle; NC: negative control. The data were analyzed using a one-way ANOVA followed by a Newman-Keuls post hoc test (p < 0.05).

The effects of the aqueous extract of *L. gerberiformis* leaves on GSH levels are summarized in Fig. 9. Mice in the negative control group showed significantly decreased [F (2, 9) = 8.870 P = 0.0074] levels of GSH in the hippocampus compared with the vehicle group.

Furthermore, the extract increased GSH levels at all doses: 75 mg/kg [F (2, 9) = 18.19,P = 0.0007], 150 mg/kg [F (2, 9) = 8.884, P = 0.0074], and 300 mg/kg [F (2, 9) = 7.755, P = 0.0110] in the hippocampus compared with the negative control group. Memantine also led to a significant increase [F (2, 9) = 5.933, P = 0.0227] in GSH levels in the hippocampus compared to the negative control group (Fig. 9a).

It appears that daily exposure of mice to AlCl_3_ for six weeks resulted in a significant decrease [F (2, 9) = 24.98, P = 0.0002] in the level of GSH in the prefrontal cortex compared to the vehicle group. Pretreatment with the aqueous extract of *L. gerberiformis* leaves at the doses of 75 mg/kg [F(2, 9) = 32.58, P < 0.0001], 150 mg/kg [F (2, 9) = 45.36,P < 0.0001], and 300 mg/kg [F (2, 9) = 13.51, P = 0.0019] showed a significant increase of GSH level compared to the negative control group (Fig. 9b). Finally, memantine also led to a significant increase [F(2, 9) = 102.0, P < 0.0001] in GSH levels in the prefrontal cortex compared with the negative control group. Compared with memantine, a dose of 75 mg/kg significantly increased [F (2, 9) = 4.696, P = 0.0401] GSH concentration in the prefrontal cortex.

### Effect of *L. gerberiformis* aqueous leaf extract on MDA and NO in the hippocampus and prefrontal cortex of mice exposed to AlCl_3_

**Fig. 10:**
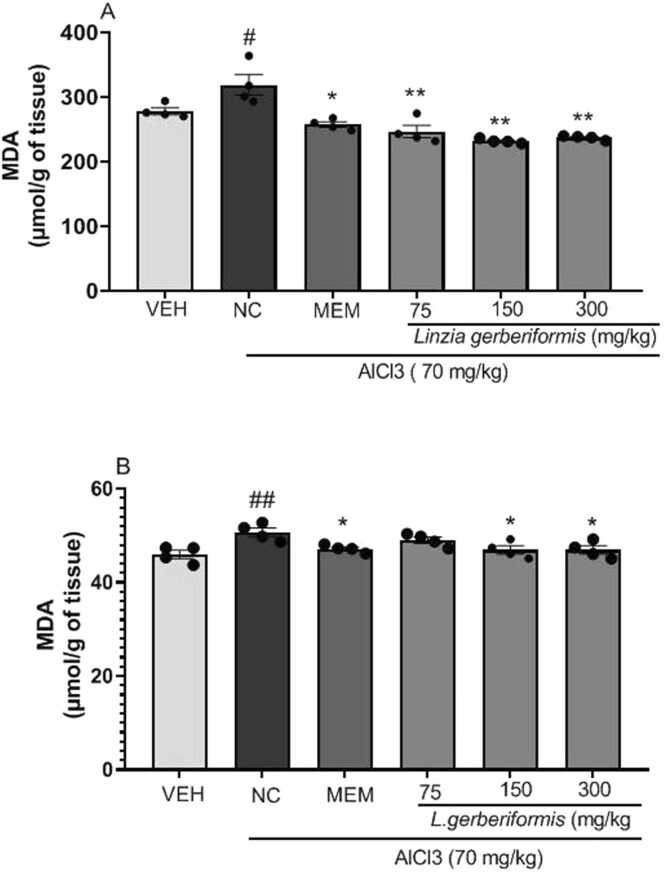
Effect of *L. gerberiformis* aqueous extract on MDA levels. A: Hippocampus; B: MDA prefrontal cortex. Each bar represents the mean ± SEM (n = 4). *P < 0.05, **P < 0.01, ***P˂0.001: significant difference from negative control. #p < 0.05; ##P < 0.01; ###P < 0.001 significant difference compared to vehicle. VEH: vehicle; NC: negative control. MEM: Memantine (20 mg/kg). The data were analyzed using a one-way ANOVA followed by a Newman-Keuls post hoc test (p < 0.05).

**Fig. 11:**
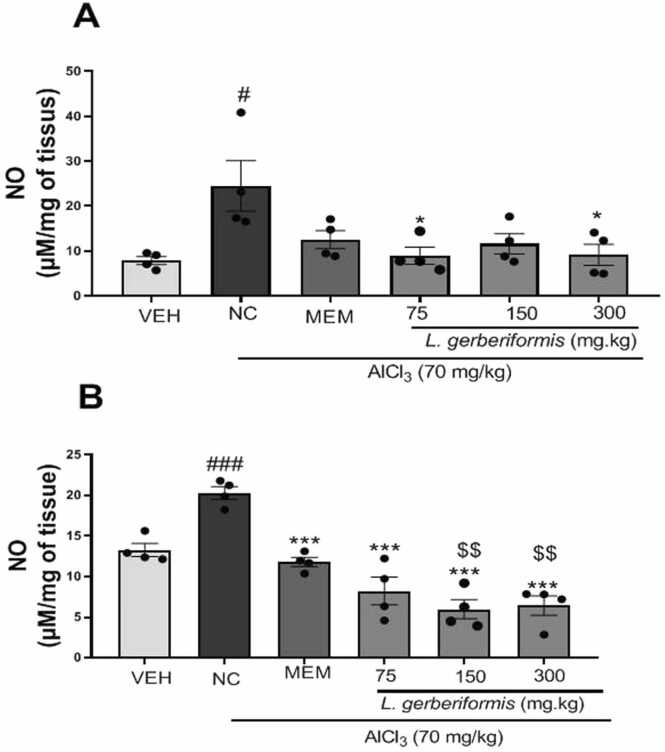
Effect of *L. gerberiformis* aqueous extract on NO levels. A: Hippocampus; B: NO prefrontal cortex. Each bar represents the mean ± SEM (n = 4). *P < 0.05, **P < 0.01, ***P˂0.001: significant difference compared to negative control. #p < 0.05; ##P < 0.01; ###P < 0.001: significant difference compared to vehicle. $$P < 0.01: significant difference compared to MEM.VEH: vehicle; NC: negative control. MEM: Memantine (20 mg/kg). The data were analyzed using a one-way ANOVA followed by a Newman-Keuls post hoc test (p < 0.05).

The results of the study on the effect of the aqueous extract of *L. gerberiformis* leaves on malondialdehyde (MDA) and Nitric Oxide (NO) levels are presented in Fig. 10, Fig. 11. Mice in the negative control group showed a significant increase in MDA levels [F(2, 9) = 5.383, P = 0.0290] in the hippocampus compared to the vehicle group. Animals pretreated with the aqueous extract of *L. gerberiformis* leaves at the doses of 75 mg/kg [F (2, 9) = 11.97, P = 0.0029], 150 mg/kg [F (2, 9) = 29.67, P = 0.0001], and 300 mg/kg [F (2, 9) = 26.35, P = 0.0002] showed a significant decrease in MDA levels in the hippocampus compared to the negative control group. Memantine administered at the dose of 20 mg/kg also showed significant [F (2, 9) = 13.35, P = 0.0020] effects, resulting in decreased MDA levels in the hippocampus compared to the negative control group (Fig. 10a).

The level of MDA in the prefrontal cortex was significantly increased [F (2, 9) = 9.320 P = 0.0064] in animals from the negative control group compared to the vehicle group. The aqueous extract of *L. gerberiformis* leaves significantly decreased the MDA levels in the prefrontal cortex at doses 150 mg/kg [F (2, 9) = 5.869, P = 0.0234] and 300 mg/kg [F (2, 9) = 5.869 P = 0.0234] compared to the negative control. Memantine administered at a dose of 20 mg/kg also showed significant effects, resulting in decreased MDA levels [F (2, 9) = 10.87, P = 0.0040] in the prefrontal cortex compared to the negative control (Fig. 10b).

It appears that daily exposure to AlCl_3_ for six weeks resulted in a significant increase [F (2, 9) = 4.283, P = 0.0493] of the level of NO in the hippocampus compared to the vehicle group.

Pretreatment with *L. gerberiformis* at the doses of 75 mg/kg [F (2, 9) = 6.189, P = 0.0204], and 300 mg/kg [F (2, 9) = 5.446, P = 0.0282] showed a significant decrease in its level compared to the negative control group (Fig. 11a). Memantine reduced NO levels in the hippocampus [F (2, 9) = 3.570, P = 0.0722] compared to the negative control group (Fig. 11a).

Mice in the negative control group showed a significant increase [F (2, 9) = 25.70, P = 0.0002] in NO levels in the prefrontal cortex compared to the vehicle group. In contrast, the extract at all doses (75 mg/kg [F (2, 9) = 22.51, P = 0.0003], 150 mg/kg [F (2, 9) = 59.51, P < 0.0001], 300 mg/kg [F (2, 9) = 54.65, P < 0.0001]) led to a significant restoration of NO levels in the prefrontal cortex compared with the negative control group. In addition, memantine reduced NO levels in the prefrontal cortex [F (2, 9) = 56.58, P < 0.0001] compared with the negative control group (Fig. 11b). At doses of 75, 150, and 300 mg/kg, the extract exhibited a nonsignificant decrease in NO compared with memantine (20 mg/kg) in the hippocampus only [F(3,12) = 0.6984, p = 0.5708]. In the prefrontal cortex, however, NO levels decreased significantly at 150 mg/kg [F(2,9) = 10.79, p = 0.0041] and 300 mg/kg [F(2,9) = 8.968, p = 0.0072] compared with memantine (20 mg/kg) (Fig. 11a and b).

## Discussion

This study assessed the effect of the aqueous extract of *L. gerberiformis leaves* on neurotoxicity-induced memory impairment using the Object Location Test (OLT) and T-maze test. The ability of AlCl_3_ to induce neurotoxicity, mimicking a key component of AD, has been well established. Once in the systemic circulation, AlCl_3_ crosses the blood-brain barrier and accumulates in the brain, particularly in areas associated with memory and learning, such as the prefrontal cortex and hippocampus. Ultimately, it generates AD-like pathology (Ekundayo et al. 2022). Previous animal studies suggested that AlCl_3_ induces cognitive impairment, oxidative stress, and cholinergic dysfunction, leading to Alzheimer's-like symptoms (Nisa et al., 2021, Firdaus et al., 2022). In our study, we used the OLT and T-maze to evaluate working memory and learning deficits in mice exposed to AlCl_3_. The OLT model exhibited a notable reduction in discrimination index compared to the vehicle, which is directly associated with hippocampal-dependent spatial memory (Ennaceur et al., 1997, Goodman et al., 2021, Ngoupaye et al., 2017). Similarly, the T-maze has demonstrated its ability to display deficits in working memory and spatial learning dependent on the hippocampus and prefrontal cortex (Deacon and Rawlins, 2006).

In view of the results obtained, the aqueous extract of *L. gerberiformis* leaves, at doses of 75 and 150 mg/kg, along with memantine (20 mg/kg), increased exploration time in the novel location, discrimination index in the location test, and percentage of correct choices in the T-maze in animals treated with AlCl_3_ (70 mg/kg). The extract's ability to prevent the neurotoxicity that leads to amnesia induced by AlCl_3_ in the novel object location test and the T-maze suggests that the extract possesses anti-amnesic properties in these behavioral tests. The presence of secondary metabolites in the extract may enhance cognitive function by mitigating oxidative stress and inflammation in the brain. These processes are known to contribute to the neurodegeneration associated with aluminium exposure (Shunan et al., 2021, Ekundayo et al., 2022). Thus, we suggest that the extract enhances learning and spatial memory processes dependent on the prefrontal cortex and hippocampus, likely through the activities of its secondary metabolites. To establish the implications of AlCl_3_ exposure on biochemical alterations that could confirm the behavioral studies, the effect of the extract on several biochemical parameters was assessed.

The brain consumes a considerable amount of oxygen to maintain its function, rendering this organ susceptible to oxidative stress (Trofin et al., 2025). This exaggerated consumption of molecular oxygen could lead to the production of a superoxide free radical anion (O_2_^-^) during mitochondrial respiratory chain reactions (Konno et al., 2021, Trofin et al., 2025). In the context of oxidative stress within living cells, a shift in the balance occurs, with endogenous antioxidants such as superoxide dismutase (SOD), catalase, and glutathione (GSH) being utilized to counteract the surge in reactive oxygen species (ROS) (Marzouk and Farid, 2023, Olufunmilayo et al., 2023, Jomova et al., 2024). The enzyme superoxide dismutase (SOD) catalyzes the dismutation of the O_2_^-^ anion into hydrogen peroxide (H_2_O_2_) and molecular oxygen (O_2_). Subsequently, the enzyme catalase functions by hydrolyzing H_2_O_2_ into H_2_O and O_2_ (Shadfar et al., 2023, Das, 2023). As indicated by Adelodun et al. (2021) and Gęgotek and Skrzydlewska (2024), biological membrane macromolecules, including proteins and lipids, are also a primary target of ROS. This phenomenon is exemplified by the elevated tissue levels of malondialdehyde (MDA), which in this instance signifies the culminating molecule in the lipid peroxidation process (Holubiec et al., 2022, Gangarde et al., 2025). Furthermore, nitric oxide (NO) functions as a neurotransmitter molecule in the central nervous system (CNS). However, when its production exceeds a certain threshold, it reacts with oxygen ions (O_2_^-^) to form peroxynitrite (ONOO^-^) at the origin of protein adducts (Ben Saad et al., 2023, Salvagno et al., 2024). The results of the present study demonstrate that, in comparison with mice exposed to AlCl_3_ (70 mg/kg) over a 42-day period, mice that were pretreated with aqueous extract of *L. gerberiformis* leaves extract exhibited a significant increase in SOD, GSH, and catalase levels in the hippocampus and prefrontal cortex, respectively. However, the administration of AlCl_3_ (70 mg/kg) to mice prior to the experiment resulted in a significant decrease in SOD, catalase, and GSH levels in the hippocampus and prefrontal cortex. This decline was concomitant with an escalation of the MDA and NO levels in the hippocampus and prefrontal cortex of mice treated with AlCl_3_ (70 mg/kg). Indeed, an increase in MDA-induced lipid peroxidation has been observed to result in degeneration of several neurons in the central nervous system, notably cholinergic and GABAergic neurons (Olayinka et al., 2022, Pekdemir et al., 2024). This phenomenon has been shown to lead to learning and memory failure in animals treated with AlCl_3_. The aqueous extract of *L. gerberiformis* leaves significantly reduced MDA levels in the hippocampus and prefrontal cortex of treated animals, compared with the negative control, showing the plant's inhibition of lipid peroxidation, suggesting its antioxidant properties. The observed decrease in NO levels indicates that the extract operates through the antioxidant pathway, trapping reactive nitrogen species, such as peroxynitrite. In vitro results demonstrate that the aqueous extract of *L. gerberiformis* leaves exhibits a 71.88 % DPPH radical inhibition and an antioxidant capacity of 89.76 mmol FeSO_4_/g, thereby substantiating the antioxidant activity observed in vivo. The antioxidant activity exhibited by these plants is likely attributable to the presence of secondary metabolites, including flavonoids, alkaloids, tannins, and phenolic acids, which function through electron transfer mechanisms. As demonstrated in previous studies, the presence of flavonoids, alkaloids, tannins, steroids, and saponins has been identified in the aqueous extract of *L. gerberiformis* leaves (Kitonde et al., 2013, Koto-te-NyiwaNgbolua et al., 2016, Wanna et al., 2023). In the DPPH assay, these compounds neutralize free radicals by donating an electron, while in the FRAP assay, they reduce ferric ions (Fe³⁺) to ferrous ions (Fe²⁺), contributing to their antioxidant activity (Huang et al. 2014). These results suggest that the anti-amnesic effect of the aqueous extract of *L. gerberiformis leaves* is the result of the inhibition of AlCl_3_-induced oxidative stress in the hippocampus and prefrontal cortex.

As postulated by Pekdemir et al. (2024), one of the consequences of lipid peroxidation in the brain is the increased production of acetylcholinesterase. Acetylcholinesterase (AChE) is responsible for the degradation of acetylcholine (ACh) into acetate and choline in the synaptic cleft of neurons in the prefrontal cortex and hippocampus. This process enables effective control of memory and learning processes in these areas (Ngoupaye et al., 2017, Ekundayo et al., 2022). As demonstrated by Ekundayo et al. (2022), the suppression of AChE synthesis has been observed to result in an augmentation of the acetylcholine (ACh) concentration within the synaptic cleft. This, in turn, has been shown to promote the stimulation of cognitive functions. In the present study, the aqueous extract from the leaves of *L. gerberiformis* exhibited a decrease in acetylcholinesterase activity and an increase in acetylcholine levels, with effects analogous to those of memantine, an NMDA receptor inhibitor. The present findings are consistent with those of Ordoñez et al. (2025), who demonstrated that the extract of *Caliphruria subedentata* (Amaryllidaceae) contains alkaloids that interact with key elements (AChE, BuChE, NMDA receptor) implicated in Alzheimer's disease (AD). Moreover, research has demonstrated that flavonoids possess inhibitory properties on acetylcholinesterase (AChE), thereby increasing acetylcholine levels (Cichon et al., 2024). The findings of this study suggest a potential beneficial effect of the aqueous extract from the leaves aqueous extract of *L. gerberiformis* leaves, which contains a variety of secondary metabolites identified by Kitonde et al. (2013). These secondary metabolites have been shown to possess anti-cholinesterase activity, which may contribute to improvements in impaired learning and long-term memory, as evidenced by studies conducted by Hampel et al. (2019) and Ngoupaye et al. (2020). These results provide further evidence to support the conclusions drawn from the behavioral studies, thereby confirming the initial hypothesis.

It is imperative to acknowledge the significance of GABA within the central nervous system, where it functions as a pivotal inhibitory neurotransmitter. GABA modulates and regulates neural excitation initiated by glutamate, as evidenced by research conducted by Almutairi et al., 2024. Additionally, GABA plays a crucial role in the learning process, as highlighted in the study by Dinse, 2023. AlCl_3_ has been demonstrated to induce severe memory impairments through the overproduction of glutamate and a concomitant decrease in GABA, which may contribute to the emergence of behavioral and psychiatric symptoms characteristic of AD (Kazmi et al. 2024). In the present study, AlCl_3_-induced neurotoxicity in rats was found to decrease GABA levels in the hippocampus and prefrontal cortex of mice. It was observed that pretreatment with the extract at all doses (75, 150, and 300 mg/kg) prevented a decrease in GABA concentration in the targeted tissue. These results align with those reported by Adelakin et al. (2024), who demonstrated that treatment with *Bryophyllum pinnatum* methanolic extract and its flavonoid-rich fraction prevented a decline in GABA levels. The synergistic effects of phenolic compounds may provide a plausible explanation for the observed enhancements in cognitive functions and long-term memory. Indeed, as suggested by Ngoupaye et al. (2020) and Pritam et al. (2022), polyphenols have been utilized for the prevention and treatment of cognitive diseases, a property that can be attributed to their antioxidant characteristics. Our findings indicate that the 150 mg/kg dose of *L. gerberiformis* exhibited the most significant neuroprotective activity when compared to the standard treatment of Memantine (20 mg/kg). Specifically, at this dose, we observed notable improvements in various measured parameters, including acetylcholine (ACh) and GABA levels, as well as reductions in acetylcholinesterase (AChE) activity, which is critical for maintaining optimal neurotransmission. Furthermore, the effects of *L. gerberiformis* were indeed found to be dose-dependent. As the dose increased, there was a corresponding enhancement in neuroprotective effects across the different cognitive and biochemical assessments. For instance, both 150 mg/kg and 300 mg/kg doses led to significant increases in ACh levels in the hippocampus and prefrontal cortex, with marked reductions in MDA and NO levels, indicating a robust protective effect against the neurotoxic impact of AlCl_3_.

## Conclusion

The aqueous extract of the leaves of *L. gerberiformis* exhibits neuroprotective properties by alleviating Alzheimer's-type cognitive impairment induced by AlCl_3_. These effects are attributed to cholinergic mechanisms, including a reduction in acetylcholinesterase activity and an increase in acetylcholine levels in the hippocampus and prefrontal cortex. Additionally, the extract enhances GABAergic signaling by increasing GABA levels, further supporting cognitive function. Its antioxidant properties also play a crucial role in mitigating oxidative stress. Furthermore, the extract improves both spatial and non-spatial working memory across various behavioral tasks. These findings advance the scientific understanding of the aqueous extract of *L. gerberiformis* leaves use in traditional medicine and highlight its potential as a neuroprotective therapeutic agent.

## Limitations and future directions

It would have been more informative to have beyond the biochemical results, the histological analysis to further confirm neurodegeneration or recovery after treatments with *L. gerberiformis*.

## CRediT authorship contribution statement

**Gwladys Temkou Ngoupaye:** Writing – review & editing, Supervision, Software, Resources, Project administration, Methodology, Data curation, Conceptualization. **Steve Brunel Kenfack Ngoufack:** Methodology, Investigation. **Jospin Chirac Noubouwo:** Writing – original draft, Software, Methodology, Investigation, Data curation, Conceptualization. **Blesdel Maxwell Adassi:** Resources. **King-Ghislain Gnoupa:** Resources. **Bibiane Tatiana Diebo Kom:** Resources, Investigation. **Aurelien Fossueh Foutsop:** Writing – review & editing, Resources. **Francis Bray Yassi:** Resources.

## Compliance with ethical standards

Animal experiments were carried out in accordance with the international principles of laboratory animal protection (NIH Publication 8023, revised 1996), and use of laboratory animals’ manual, and Directives 2010/63/EU for animal experiments. Efforts have been made to minimize animal suffering as much as possible.

## Consent to participate

Not applicable.

## Consent for publication

Not applicable.

## Funding

No specific grant, fund from funding agencies were receiving during this research.

## Conflicts of Interest

The authors have non-financial interests to disclose.
